# Mitotic phosphorylation of CCCTC‐binding factor (CTCF) reduces its DNA binding activity

**DOI:** 10.1002/2211-5463.12189

**Published:** 2017-01-25

**Authors:** Takeshi Sekiya, Kensaku Murano, Kohsuke Kato, Atsushi Kawaguchi, Kyosuke Nagata

**Affiliations:** ^1^ Department of Infection BiologyFaculty of Medicine and Graduate School of Comprehensive Human ScienceUniversity of TsukubaJapan; ^2^ Department of Molecular BiologyKeio University School of MedicineTokyoJapan; ^3^ Faculty of MedicineUniversity of TsukubaJapan

**Keywords:** higher order chromatin structure, mitosis, zinc finger protein

## Abstract

During mitosis, higher order chromatin structures are disrupted and chromosomes are condensed to achieve accurate chromosome segregation. CCCTC‐binding factor (CTCF) is a highly conserved and ubiquitously expressed C2H2‐type zinc finger protein which is considered to be involved in epigenetic memory through regulation of higher order chromatin architecture. However, the regulatory mechanism of CTCF in mitosis is still unclear. Here we found that the DNA‐binding activity of CTCF is regulated in a phosphorylation‐dependent manner during mitosis. The linker domains of the CTCF zinc finger domain were found to be phosphorylated during mitosis. The phosphorylation of linker domains impaired the DNA‐binding activity *in vitro*. Mutation analyses showed that amino acid residues (Thr289, Thr317, Thr346, Thr374, Ser402, Ser461, and Thr518) located in the linker domains were phosphorylated during mitosis. Based on these results, we propose that the mitotic phosphorylation of the linker domains of CTCF is important for the dissociation of CTCF from mitotic chromatin.

**Abbreviations**
ChIP: chromatin immunoprecipitation
CTCF: CCCTC‐binding factor
ZF: zinc finger

*Cis*‐acting regulatory DNA elements such as insulators and enhancers are involved in the temporal and cell type‐specific control of gene expression through the formation of higher order chromatin structure. Higher order chromatin structure is tightly regulated throughout the cell cycle to achieve proper and dynamic chromosome processes. CCCTC‐binding factor (CTCF) is a transcription factor containing 11 highly conserved zinc finger motifs (ZF1‐ZF11; Fig. 1) responsible for its DNA‐binding activity 1, 2. CTCF also mediates higher order chromatin structure formation by modulation of chromatin loops that define the boundary between active and inactive chromatin 3, 4.

**Figure 1:**
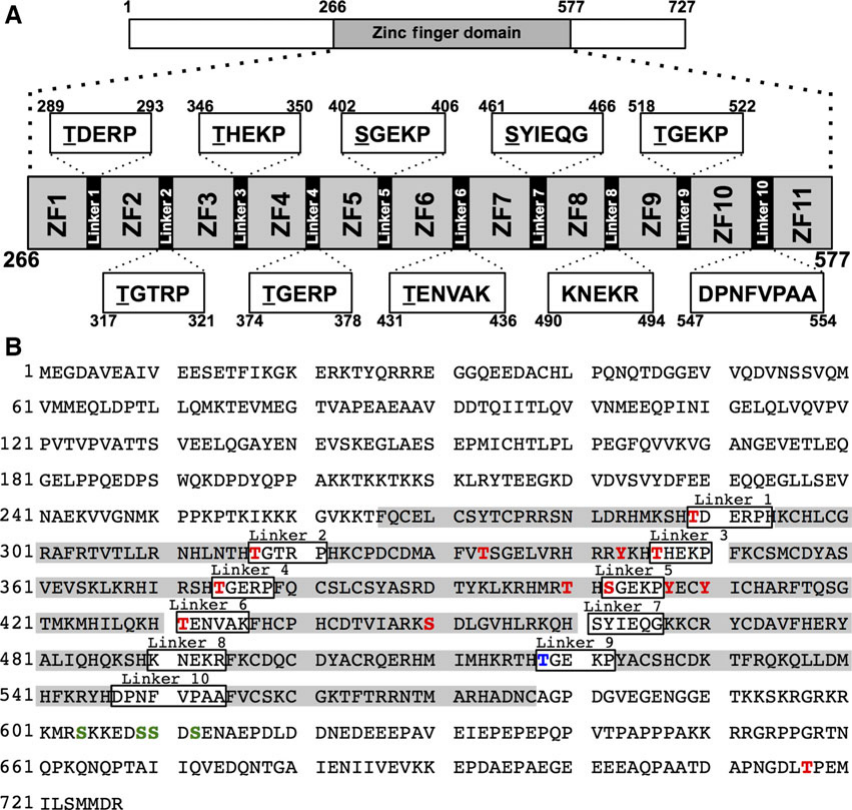
Schematic diagram of human CTCF. (A) Zinc finger domains. Amino acid sequences of each linker domain are indicated, and candidate amino acid residues for phosphorylation are underlined. (B) The amino acid sequence of human CTCF. Zinc finger motifs are highlighted in gray. All known phosphorylation amino acid positions are marked. Red characters indicate phosphorylation sites identified by proteomics analyses in 9 and 10. Blue and green characters indicate phosphorylation sites analyzed in 11 and 17, respectively.

More than 3% of the total number of human genes belongs to the zinc finger protein family. Most zinc finger proteins, including CTCF, have tandemly repeated multiple zinc finger motifs separated by a highly conserved short linker peptide sequence, TGEKP. This linker domain is important for the structural stabilization of the zinc finger motif by forming an alpha‐cap structure 5, and the DNA‐binding activity of zinc finger motifs is regulated by phosphorylation of threonine residues in the linker domain during mitosis 6, 7, 8. CTCF has 10 linker domains, but only linker domain 9 located between ZF9 and ZF10 contains the exact TGEKP sequence. It has been reported that CTCF is highly phosphorylated at multiple amino acid residues including T518 at the linker domain 9 9, 10, 11. However, the effect of mitotic phosphorylation on CTCF DNA‐binding activity with regard to each phosphorylation site is not well understood.

## Materials and methods

### Cell culture, synchronization, and transfection

HeLa S3 cells and MCF‐7 cells were maintained at 37 °C in Dulbecco's modified Eagle's medium (DMEM) supplemented with 10% fetal bovine serum. Cells were synchronized at mitotic phase by two cycles of “excess thymidine blockage followed by nocodazole arrest”. Briefly, at 12 h post treatment of 2.5 mm thymidine (Sigma‐Aldrich Co. LLC., St. Louis, MO, USA), cells were released into a fresh culture medium without an excess amount of thymidine for 10 h, and then synchronized again at G1/S boundary in growth medium with 2.5 mm thymidine for 14 h. At 6 h post release from thymidine treatment, cells were treated with 165 nm nocodazole (Sigma‐Aldrich Co. LLC.) for 6 h. Mitotic cells were collected by gentle shaking of cell culture dishes. Transient DNA transfection assays were performed using Gene Pulser Xcell System (Bio‐Rad Laboratories Inc., Hercules, CA, USA) according to the manufacturer's protocol.

### Construction of plasmids

To make 3 × FLAG‐tagged CTCF WT expression vector, CTCF cDNA fragment from pBS‐FLAG‐CTCF 12 was cloned into pCAGGS‐3 × FLAG 13. To construct 4A, T518A, 7A, 8A, and 8D expression vectors, cDNA fragments containing each point mutation were amplified by PCR using the primers shown in Table 1. 3 × FLAG‐tagged cDNA fragments were cloned into pCAGGS vector.

**Table 1:** Primers for plasmids construction

Primer Name	Sequence
T289A/D rev	cGTGGCTTTTCATGTGACG
T289A for	CTGATGAGAGACCACACAAG
T289D for	acGATGAGAGACCACACAAG
T317A/D rev	cGTGTGTGTTAAGGTGATTC
T317A for	CAGGTACTCGTCCTCACAAG
T317D for	acGGTACTCGTCCTCACAAG
T346A/D rev	cGTGTTTGTAACGACGATGC
T346A for	CCCACGAGAAGCCATTCAAG
T346D for	acCACGAGAAGCCATTCAAG
T374A/D rev	cATGAGAGCGAATGTGACG
T374A for	CTGGAGAGCGTCCGTTTCAG
T374D for	acGGAGAGCGTCCGTTTCAG
S402A/D rev	cATGGGTTCTCATGTGCC
S402A for	CAGGGGAAAAGCCTTATGAA
S402D for	acGGGGAAAAGCCTTATGAA
T431A/D rev	cGTGCTTCTGTAAAATGTGC
T431A for	CAGAAAATGTGGCCAAATTTC
T431D for	acGAAAATGTGGCCAAATTTC
S461A/D rev	cATGCTGCTTTCGCAAGTGG
1382A for	CCTATATTGAGCAAGGCAAG
1382D for	acTATATTGAGCAAGGCAAG
T518A/D rev	cGTGGGTGCGCTTGTGCATG
T518A for	CCGGGGAGAAGCCTTACGCC
T518D for	acGGGGAGAAGCCTTACGCC
S4A rev	ATCTTCTTTCTTAGcGCGCATCTTTC
S4A for	gCCgCTGACgcTGAAAATGCTGAACCAGATC

### Antibodies

A rabbit polyclonal anti‐CTCF antibody was prepared as previously described 12. Anti‐FLAG (M2, Sigma‐Aldrich Co. LLC.), rabbit polyclonal anti‐histone H3 (ab1791, Abcam plc., Cambridge, UK) and anti‐phospho‐histone H3 antibodies (06‐570, Merck Millipore, Billerica, MA, USA) were purchased.

### Subcellular fractionation

Cells were washed with cold PBS(−), and kept on ice for 5 min in a cold hypotonic buffer (10 mm HEPES‐NaOH, pH 8.0, 10 mm KCl, 1.5 mm MgCl_2_, 0.1% Triton X‐100, 5 mm Na_3_VO_4_, 10 mm NaF, 25 mm glycerol 2‐phosphate) on ice for 5 min. Supernatant and pellet fractions were obtained by centrifugation at 2300 ***g*** for 5 min.

### ChIP assay

Cells were washed with cold PBS(−), and kept on ice for 5 min in a cold CSK buffer (10 mm PIPES‐NaOH, pH 6.8, 100 mm NaCl, 3 mm MgCl_2,_ 1 mm EGTA, 300 mm sucrose, 0.1% Triton X‐100, 1 mm Na_3_VO_4_, 1 mm NaF, 5 mm glycerol 2‐phosphate). After centrifugation at 2300 ***g*** for 5 min, pellet fractions were incubated at 25 °C for 10 min in CSK buffer containing 0.5% formaldehyde, and then incubated with 25 °C for 5 min in 0.125 m glycine/PBS. Immunoprecipitation assays were carried out according to the manufacturer's protocol (Chromatin Immunoprecipitation Assay Kit; Merck Millipore). The quantitative PCR (qPCR) was performed with primers 5′‐GGTCCACGGGCCGCCCTGCCAG‐3′ and 5′‐CGCAGCTCCGGAAGCCGAGAGC‐3′ corresponding to a part of *rRNA* gene between nucleotide positions −961 and −851, where +1 is set to be the transcription start site 14 using FastStart SYBR Green Master (Roche Molecular Systems, Inc., Hacienda, CA, USA) and Thermal Cycler Dice Real Time System (TaKaRa Bio Inc., Shiga, Japan) according to the manufacturers’ protocols.

### DNA‐binding assay

Asynchronously growing MCF‐7 cells were incubated on ice for 5 min in the hypotonic buffer. Supernatant and pellet fractions were separated by centrifugation at 2300 ***g*** for 5 min, and the pellet fractions were incubated on ice for 5 min in a buffer (10 mm Tris/HCl, pH 7.9, 275 mm NaCl, 1 mm MgCl_2_, 0.1% Triton X‐100, 1 mm Na_3_VO_4_, 1 mm NaF, 5 mm glycerol 2‐phosphate). Nocodazole‐arrested MCF‐7 cells were incubated on ice for 5 min in the hypotonic buffer. CTCF was immunoprecipitated from the lysates using the anti‐CTCF antibody. After washing with a buffer containing 10 mm Tris/HCl, pH 7.9, 500 mm NaCl, 1 mm MgCl_2_, 0.1% Triton X‐100, 1 mm Na_3_VO_4_, 1 mm NaF, 5 mm glycerol 2‐phosphate, immunoprecipitated proteins were incubated with or without lambda protein phosphatase (P7053, New England Biolabs Inc., Ipswich, MA, USA) at 30 °C for 2 h, and then incubated in a DNA‐binding buffer (20 mm Tris/HCl, pH 7.4, 150 mm NaCl, 2 mm MgCl_2_, 0.01% NP‐40, 6.25% glycerol, 1 mm Na_3_VO_4_, 1 mm NaF, 5 mm glycerol 2‐phosphate) with 0.15 ng of the *rRNA* gene upstream region fragment and 50 ng of poly (dIdC). The *rRNA* gene upstream region fragment used here is amplified from genomic DNA and purified from agarose gel. The exact sequence of the fragment corresponding to the region between nucleotide positions −961 and −851, where +1 is set to be the transcription start site 14 is as follows: 5′‐ GGTCCACGGGCCGCCCTGCCAGCCGGATCTGTCTCGCTGACGTCCGCGGCGGTTGTCGGGCTCCATCTGGCGGCCGCTTTGAGATCGTGCTCTCGGCTTCCGGAGCTGCG‐3′, where the CTCF‐binding site is underlined. The amounts of coimmunoprecipitated DNA were analyzed by qPCR and normalized by the protein amount of CTCF measured by imagej software (developed at the National Institutes of Health, Bethesda, MD, USA) from western blotting results.

## Results

### CTCF is dissociated from mitotic chromatin

**Figure 2:**
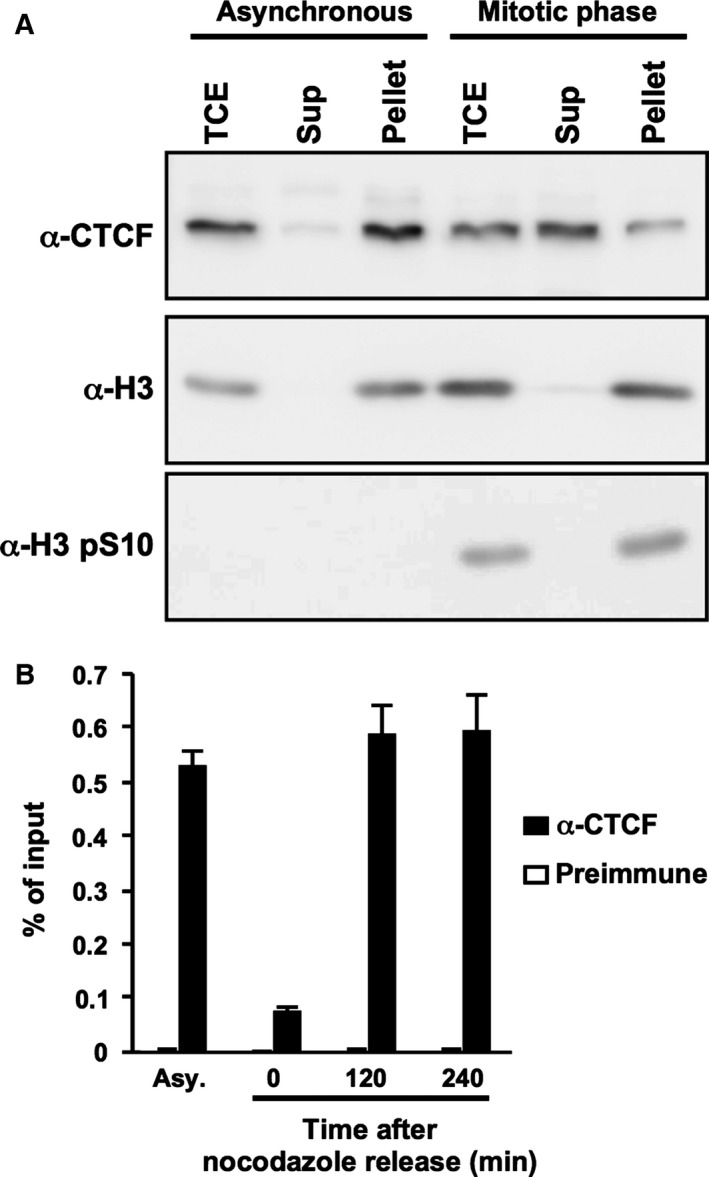
CTCF is dissociated from mitotic chromatin. (A) Fractionation of asynchronous and nocodazole‐arrested HeLa S3 cells. Total cell extracts (TCE), and supernatant and pellet fractions were subjected to western blotting using anti‐CTCF, anti‐histone H3, and anti‐phospho‐histone H3 antibodies. Phosphorylated histone H3 was used as a marker of the mitotic chromatin fraction. (B) Chromatin‐binding activity of mitotic CTCF. HeLa S3 cells were collected at 2 or 4 h post release from nocodazole treatment. The cell lysates were subjected to ChIP assays with anti‐CTCF antibody (filled bars) or rabbit preimmune serum (open bars). qPCR was performed with primers specific for the upstream region of the *rRNA* gene. The amount of DNA coprecipitated with each antibody was shown as % of input.

It is reported that most of the C2H2 zinc finger family proteins are excluded from mitotic chromosomes 11. To determine the amount of CTCF bound to mitotic chromatin, subcellular fractionation was performed using a hypotonic buffer. HeLa S3 cells were synchronized at mitosis as described above. The amount of CTCF in the supernatant fraction obtained from mitotic cells was increased compared to asynchronous cells (Fig. 2A). It has been reported that CTCF binds upstream of the *rRNA* gene promoter 14, so that next we examined the amount of CTCF bound to the *rRNA* gene locus during mitosis by chromatin immunoprecipitation (ChIP) assays. The amount of CTCF on the *rRNA* gene locus in mitotic cells was 80% less than that in asynchronous cells (Fig. 2B). After release from the mitotic block, the amount of CTCF on the *rRNA* gene was restored (Fig. 2B). These results suggest that CTCF is dissociated from chromatin during mitosis and reassociated upon G1 entry.

### Phosphorylation of CTCF in mitosis

**Figure 3:**
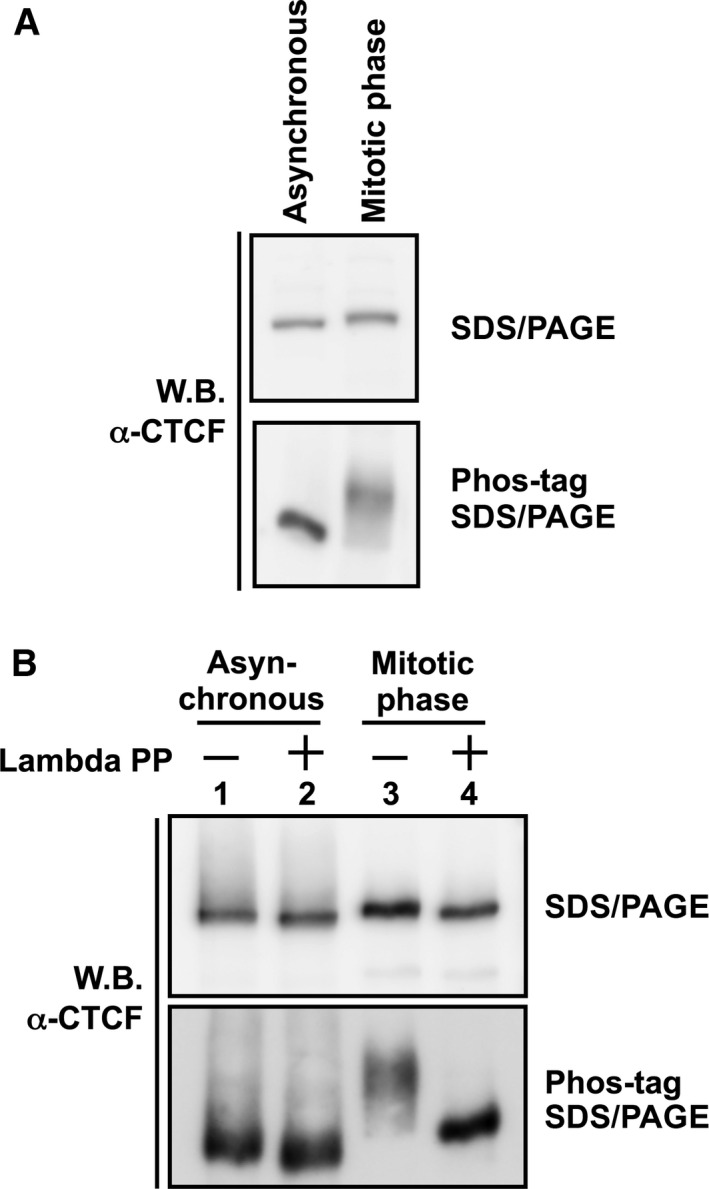
Phosphorylation of mitotic CTCF. (A) Analysis by phos‐tag SDS/PAGE. Whole cell lysates were separated on a 7.5% SDS/PAGE (upper panel) and a 5% SDS/PAGE containing 20 μm phos‐tag (lower panel), followed by western blotting with anti‐CTCF antibody. (B) Lambda phosphatase treatment of mitotic CTCF. Cell lysates prepared from asynchronous cells (lane 1) or mitotic‐arrested cells (lane 2) were subjected to immunoprecipitation assays using anti‐CTCF antibody. The immunoprecipitated CTCF was incubated in the absence or presence of lambda protein phosphatase and subjected to phos‐tag SDS/PAGE as described above.

It has been reported that a variety of DNA‐binding factors including transcription factors are phosphorylated and released from highly condensed chromosomes during mitosis 15. To examine whether CTCF is also phosphorylated in the mitotic phase, we carried out phos‐tag SDS/PAGE 16 which relies on the fact that phosphorylated proteins migrate slower than unphosphorylated proteins in the phos‐tag gel. CTCF in mitotic cell lysates migrated slower than that in asynchronous cell lysates on phos‐tag SDS/PAGE (Fig. 3A), suggesting that mitotic CTCF is phosphorylated and is therefore slower to migrate. To confirm the phosphorylation of CTCF during mitosis, the immunoprecipitated CTCF was treated with lambda protein phosphatase and subjected to phos‐tag electrophoresis. The phosphatase treatment resulted in the increased migration rate of mitotic CTCF (Fig. 3B, lanes 3, 4). These results suggest that CTCF is phosphorylated during mitosis. Both mitotic and asynchronous CTCF showed a slight increase in migration rate after lambda protein phosphatase treatment (Fig. 3B, lanes 1, 2). This result suggests phosphorylation of CTCF in interphase. It has been reported that the amino acid residues (Ser604, Ser609, Ser610, Ser612) of the C‐terminal domain of CTCF are phosphorylated by CK2 17. Phosphorylation of these amino acid residues, especially Ser612, is involved in functional switching of CTCF from transcriptional repressor to activator in *c‐myc* 18. Thus, CTCF is phosphorylated in not only mitosis but also interphase.

### The linker domains of CTCF zinc finger domain are phosphorylated during mitosis

**Figure 4:**
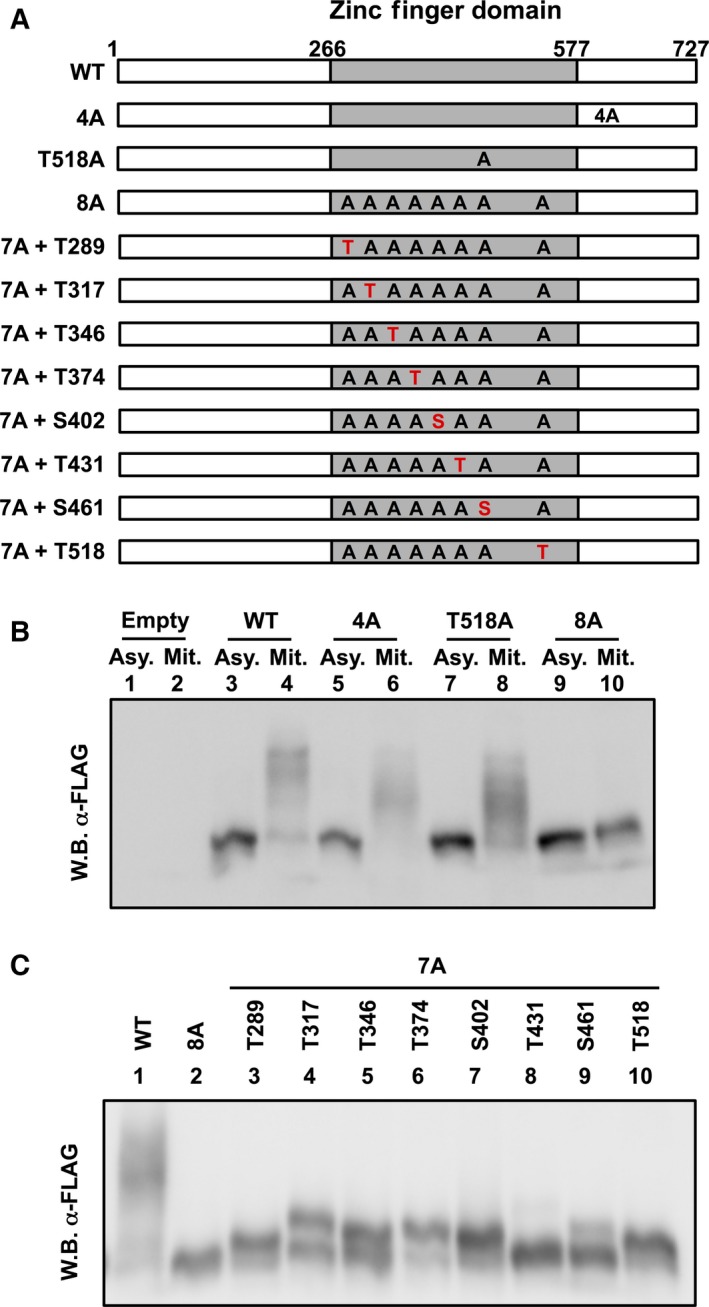
The linker domains of CTCF zinc finger domain are phosphorylated during mitosis. (A) Schematic representation of the CTCF mutants. The gray boxes indicate the zinc finger domains. 4A mutant has S604A, S609A, S610A, and S612A mutations. 8A mutant has T289A, T317A, T346A, T374A, S402A, T431A, S461A, and T518A mutations. 7A mutants have alanine substitutions in seven of eight amino acid residues, respectively. (B) Phosphorylation of exogenously expressed CTCF mutants by phos‐tag SDS/PAGE. At 72 h post transfection of CTCF mutants, asynchronously cultured or nocodazole‐arrested HeLa S3 cells were subjected to phos‐tag SDS/PAGE, followed by western blotting with anti‐FLAG M2 antibody. (C) At 72 h post transfection of CTCF mutants, nocodazole‐arrested HeLa S3 cells were subjected to phos‐tag SDS/PAGE, followed by western blotting with anti‐FLAG M2 antibody.

It has been reported that serine residues at amino acid positions 604, 609, 610, and 612 of CTCF are phosphorylated by protein kinase CK2 17. Thr518, which is located in the linker domain 9, is phosphorylated during mitosis 11. Thus, we constructed alanine‐substituted mutants of these respective amino acid residues (Fig. 4A; designated 4A and T518A). Similar to the results of 3 × FLAG‐CTCF WT (Fig. 4B, lanes 3 and 4), ectopically expressed 3 × FLAG‐CTCF 4A and T518A were still phosphorylated during mitosis (Fig. 4B, lanes 5–8), suggesting that these residues are not critical as a mitotic phosphorylation signal. To determine the location of the phosphorylated amino acid residues of CTCF during mitosis, we substituted serine and threonine residues at amino acid positions 289, 317, 346, 374, 402, 431, 461, and 518 of the linker domains (8A mutant, Figs 1 and 4A). 3 × FLAG‐CTCF 8A in mitotic cells migrated similarly to asynchronous cells (Fig. 4B lanes 9 and 10), suggesting that some of these amino acid residues are phosphorylated during mitosis. Furthermore, serine residues at amino acid position 604, 609, 610, and 612 are not phosphorylated in mitosis. Next, to identify the phosphorylated amino acid residues of linker domains in mitosis, we introduced respective alanine substitutions to seven of eight amino acid residues. Among them, only the 3 × FLAG‐CTCF 7A + T431 mutant shows the same migration rate as 3 × FLAG‐CTCF 8A mutant (Fig. 4C lanes 2 and 8). These results strongly suggest that each candidate amino acid residue of the linker domains except for T431 is phosphorylated during mitosis.

### Mitotic phosphorylation of CTCF decreases the DNA‐binding activity

**Figure 5:**
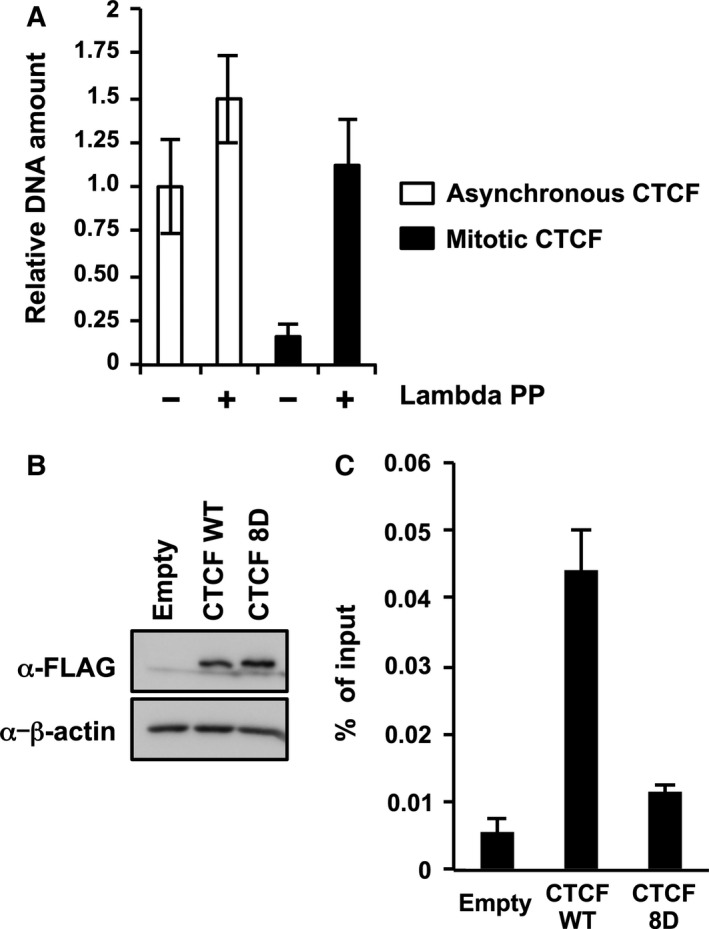
The DNA‐binding activity of CTCF is regulated by phosphorylation of linker domains during mitosis. (A) *In vitro* DNA‐binding assays. Immunoprecipitated CTCF prepared from asynchronous cell extracts or mitotic cell extracts were treated in the absence or presence of lambda protein phosphatase, and then incubated with the DNA fragments of the *rRNA* gene upstream region between nucleotide positions −60 and −851. The amount of coimmunoprecipitated DNA fragments was quantified by qPCR and normalized with the amount of CTCF protein. The mean value and standard deviations determined from three independent experiments are shown. (B) Expression level of exogenous CTCF in transfected cells. HeLa S3 cells were transiently transfected with plasmids expressing either CTCF WT or 8D mutant. At 72 h post transfection, cell lysates were subjected to SDS/PAGE, followed by western blotting. (C) Chromatin‐binding activity of CTCF phosphomimetic mutant. The cell lysates were subjected to ChIP assays with anti‐FLAG M2 antibody. qPCR was performed with primers specific for the upstream region of the *rRNA* gene. The amount of DNA coprecipitated with each antibody was shown as % of input. The mean value and standard deviations determined from three independent experiments are shown.

To determine whether the mitotic phosphorylation of linker domains affects the DNA‐binding activity, CTCF was purified using anti‐CTCF antibody from either asynchronous or mitotic MCF‐7 cell extracts and subjected to *in vitro* DNA‐binding assays. The purified CTCF, bound to protein A agarose beads, was treated with or without lambda protein phosphatase, and incubated with a fragment of the *rRNA* gene that contains the CTCF‐binding site. Quantification of fragments that interacted with CTCF was accomplished by qPCR and normalized against the protein amount of CTCF that was eluted from protein A agarose beads and quantified by western blotting. The DNA‐binding activity of mitotic CTCF was 80% lower than asynchronous CTCF (Fig. 5A). However, phosphatase treatment restored the DNA‐binding activity of mitotic CTCF (Fig. 5A). These results indicate that CTCF decreases its DNA‐binding activity in a phosphorylation‐dependent manner during mitosis. Next, we examined the DNA‐binding activity of a phosphomimetic mutant, termed CTCF‐8D, in which eight putative mitotic phosphorylation sites were changed to aspartic acid. 3 × FLAG‐tagged WT and 8D mutants of CTCF gave very similar expression levels in HeLa S3 cells (Fig. 5B). To analyze the DNA‐binding activity of the phosphomimetic mutant of CTCF, we carried out ChIP assays of exogenously expressed CTCF. The amount of the CTCF‐binding site in the *rRNA* gene locus bound to 3 × FLAG‐CTCF 8D was reduced by 30% compared to wild‐type (Fig. 5C). These results suggest that the mitotic phosphorylation of CTCF decreases its DNA‐binding activity.

## Discussion

It has been reported that the phosphorylation of linker domains reduces the DNA‐binding activity of C2H2 zinc finger proteins 6, 7, 8. Structural analyses indicate that the threonine residues of linker domains are required for the stabilization of the interaction between DNA and zinc finger proteins by providing the capping structure for the alpha‐helix of the zinc finger motif 5. Our results suggest that the DNA‐binding activity of CTCF is similarly regulated by the phosphorylation of linker domains during mitosis. It has been reported that serine/threonine kinases TOPK/PBK 19 and Cdk1 20 phosphorylate the threonine residues of the conserved linker domains. Compared with other phosphorylated residues, the phosphorylation level of Ser461 in the linker domain 7 was weak, but a significant amount of band shift was observed during mitosis (Fig. 4). The peptide sequence of the linker domain 7 is different from the consensus motif (Fig. 1). Thus, it is possible that the DNA‐binding activity of CTCF is regulated by an unknown protein kinase in addition to TOPK/PBK and Cdk1.

It has been proposed that mitotic chromosomes exist in a highly condensed state to ensure stable and integral chromosome segregation 21. Because transcription‐related factors are excluded from mitotic chromatin, mitotic chromosomes are transcriptionally inactive 15. In contrast, some markers for epigenetic gene control are retained during mitosis 22. It is possible that transcription and the formation of higher order chromatin structures mediated by CTCF might result in a disadvantage to achieve accurate chromosome segregation; however, we found that a part of CTCF still interacts with mitotic chromatin as reported previously (Fig. 2A) 23, 24. It is known that CTCF recognizes a wide variety of target gene loci using various combinations of the zinc finger domains 25, 26. We found that mitotic CTCF is detected on phos‐tag SDS/PAGE as a smeared migration pattern (Figs 3 and 4), suggesting that CTCF is phosphorylated heterogeneously in mitosis. Therefore, it is possible that the partial phosphorylation of CTCF allows for a partial interaction with gene loci even during mitosis, possibly for the probable bookmarking of chromatin domains to achieve proper gene expression at the subsequent G1 phase.

## Conclusions

Here we have shown that CTCF dissociates from mitotic chromosomes. Mutation analyses indicated that CTCF is phosphorylated in mitosis at Thr289, Thr317, Thr346, Thr374, Ser402, Ser461, and Thr518, all of which are located in linker domains. The mitotic phosphorylation of CTCF resulted in the reduction of the DNA‐binding activity to a CTCF‐binding site on *rRNA* upstream regions. These results suggest that the DNA‐binding activity of CTCF could be regulated through the phosphorylation of linker domains during mitosis.

## Author contributions

TS conceived the research strategies, performed experiments, analyzed the data, and wrote the manucsript. KS designed the experiments and analyzed the data. KK and AK designed the experiments, analyzed the data, and wrote the manuscript. KN supervised the research and wrote the manuscript.
